# 骨形成蛋白7通过激活Ⅰ型受体BMPR1A、BMPR1B抑制肺大细胞癌NCI-H460细胞的增殖

**DOI:** 10.3779/j.issn.1009-3419.2010.07.01

**Published:** 2010-07-20

**Authors:** 慧慧 徐, 艳丽 齐, 嵩 顿, 英 高, 雪杉 邱

**Affiliations:** 1 110001 沈阳，中国医科大学附属第四医院病理科，中国医科大学基础医学院病理教研室 Department of Pathology, the Forth Affiliated Hospital of China Medical University and Department of Pathology, College of Basic Medical Sciences, China Medical University, Shenyang 110001, China; 2 124010 盘锦，盘锦辽河油田第二职工医院病理科 Department of Pathology, the Second Staff Hospital of Liaohe Oilfield of Panjin, Panjin 124010, China; 3 110033 沈阳，96101部队卫生所 Medical Station, Army of 96101, Shenyang 110033, China; 4 110032 沈阳，中国医科大学附属第四医院病理科 Department of Pathology, the Forth Affiliated Hospital of China Medical University, Shenyang 110032, China; 5 110001 沈阳，中国医科大学基础医学院病理教研室，中国医科大学附属第一医院病理科 Department of Pathology, College of Basic Medical Sciences, China medical University and Department of Pathology, the First Affiliated Hospital of China Medical University, Shenyang 110001, China

**Keywords:** 骨形成蛋白7, 骨形成蛋白Ⅰ型受体, 细胞增殖, 肺肿瘤, Bone morphogenetic protein 7, Bone morphogenetic protein receptors type Ⅰ, Cell proliferation, Lung neoplasms

## Abstract

**背景与目的:**

已有的研究发现骨形成蛋白7（bone morphogenetic protein 7, BMP7）具有抑制和促进多种肿瘤发生发展的双重作用，但其对肺癌细胞增殖的影响及其具体机制尚不明确。本实验首先检测了外源性BMP7对肺癌细胞增殖的影响，然后通过在肺癌细胞系中阻断不同的Ⅰ型受体，观察其对BMP7生物学作用的影响，以探讨不同的Ⅰ型受体在BMP7信号传导过程中的作用。

**方法:**

应用RT-PCR及MTT方法分别检测4种非小细胞肺癌（non-small cell lung cancer, NSCLC）细胞系和人支气管上皮细胞系（HBE）中BMP7 Ⅰ型受体的表达情况及外源性BMP7对肺癌细胞增殖能力的影响，并联合运用抗体阻断的方法阻断NCI-H460细胞中内源性Ⅰ型抗体，采用MTT法检测BMP7对NCI-H460细胞增殖的影响，分析不同的Ⅰ型受体在BMP7信号传导过程中的作用。

**结果:**

NCI-H460细胞系中三种Ⅰ型受体均有表达。外源性BMP7抑制了肺大细胞癌NCI-H460细胞的增殖（*P*=0.002）。运用特异性抗体阻断NCI-H460细胞内源性BMPR1A、BMPR1B、BMPR1A+BMPR1B后BMP7对NCI-H460增殖的抑制作用明显减弱（*P*=0.003, *P*=0.014, *P* < 0.001），而阻断ACVR1A后BMP7对NCI-H460增殖的抑制作用无明显变化（*P*=0.074）。

**结论:**

BMP7通过激活BMPR1A、BMPR1B两种Ⅰ型受体抑制NCI-H460细胞的增殖。

## Abstract (en)

**Background and objective:**

It has been demonstrated that bone morphogenetic protein 7 (BMP7) may both inhibit and enhance cell proliferation of many kinds of cancers, but the impact of BMP7 on lung cancer cells and the exact mechanisms are not clear. The aim of this study is to investigate the effect of BMP7 on proliferation of lung carcinoma cells and explore the roles of different types of Ⅰ receptors in BMP7 signal transmission by blocking endogenous BMPRIs.

**Methods:**

The levels of expression of BMPIRs (BMP7 type Ⅰ receptors) mRNA in four different NSCLC (human non-small cell lung tumor) cell lines and HBE (normal human bronchial epithelial) cell were detected by RT-PCR. The responsiveness of pulmonary large carcinoma NCI-H460 cell to BMP7 treatment as well as to a combination of BMP7 and anti-BMPIRs treatment in proliferation were detected by MTT.

**Results:**

RT-PCR showed that NCI-H460 cells expressed all three types of BMPIRs; MTT showed that BMP7 inhibit the proliferation of NCI-H460 cell line. Blocking endogenous BMPR1A, BMPR1B obviously reversed the inhibition of BMP7 on the proliferation of NCI-H460 cell respectively (*P*=0.003, *P*=0.014). Moreover, blocking both endogenous BMPR1A and BMPR1B almost offset the effect of BMP7 on the proliferation of NCI-H460 cell completely (*P* < 0.001). But ACVR1A blocking did not affect the proliferation of NCI-H460 cell *et al* (*P*=0.074).

**Conclusion:**

BMP7 signaling via BMPR1A and BMPR1B inhibits the proliferation of pulmonary large carcinoma cell NCI-H460.

BMPs是TGF-beta家族成员之一，除了在骨和软骨的发育及诱导异位骨和软骨发生的过程中具有重要作用外，它们还具有调控细胞增殖、分化、凋亡等多种生物学作用^[1, 2]^。BMP家族受体分为Ⅰ型和Ⅱ型，Ⅰ型受体包括BMPR1A、BMPR1B、ACVR1A，Ⅱ型受体包括ACVRⅡA、ACVRⅡB、BMPR2^[2]^。其中Ⅰ型受体决定了信号传递的特异性^[3-5]^。近年来很多研究^[9-19]^表明BMP7具有抑制和促进多种肿瘤发生发展的双重作用。但是，BMP7对肺癌细胞增殖的影响及其究竟是通过分别激活哪几种Ⅰ型受体抑制和促进肿瘤细胞的增殖目前尚未明确。本实验首先检测了BMP7对肺癌细胞增殖的影响，随后通过在肺癌细胞系中阻断不同的Ⅰ型受体，观察其对BMP7处理后细胞增殖作用的影响，初步探讨不同的Ⅰ型受体在BMP7信号传导过程中的作用机制。

## 1 材料与方法

### 1.1 试剂

DMEM、RPMI-1640培养基购自美国Gibco BRL公司；重组人BMP7细胞因子购自R & D公司（克隆号：354-BP）；鼠单克隆抗人BMPR1A、BMPR1B、ACVR1A抗体均购自Santa Cruz公司（克隆号：sc-73750；sc-73751；sc-73676）；RT-PCR试剂盒购自Takara公司；MTT购自Amresco公司。

### 1.2 细胞培养

肺腺癌细胞A549在含10%胎牛血清的DMEM培养液中贴壁生长；人支气管上皮细胞HBE在含15%胎牛血清的RPMI-1640培养液中贴壁生长；NCI-H460、SPC-A-1、LTEP-a-2在含10%胎牛血清的RPMI-1640培养液中贴壁生长，均在37 ℃、5%CO_2_条件下培养。

### 1.3 反转录-聚合酶链式反应（rever se transcr iptase polymerase chain reaction, RT-PCR）提取细胞总RNA

按RT-PCR（TaKaRa）试剂盒方法进行逆转录反应，反应体系为20 μL，取2 μL的RT产物进行PCR反应，反应体系为20 μL，BMP7引物F：5’-GGT CAT GAG CTT CGT CGT CAA CC-3’，R：5’-GCA GGA AGA GAT CCG ATT CC-3’，片段长度：235 bp，退火温度为58 ℃；BMPR1A引物F：5’-TGA TTT GGA ACA GGA TGA AGC-3’，R：5’-TGT AGT ACA TTT CAG GAA GTC-3’，片段长度：382 bp，退火温度为56 ℃；BMPR1B引物F：5’-GAC ACT CCC ATT CCT CAT C-3’，R：5’-GCT ATA GTC CTT TGG ATC AG-3’，片段长度：355 bp，退火温度为55 ℃；ACVR1A引物F：5’-GCA TTC CCA GAG CAC CAA TC-3’，R：5’-CTG TGA GTC TTG CGG ATG GA-3’，片段长度：383 bp，退火温度为59 ℃；β-actin引物F：5’-AAA TCG TGC GTG ACA TTAA-3’，R：5’-CTC GTC ATA CTC CTG CTT G-3’，片段长度：513 bp，退火温度为55 ℃。PCR产物琼脂糖凝胶电泳进行检测，采用凝胶成像分析系统进行半定量分析。

### 1.4 生长曲线测定

以1×10^3^/孔的细胞密度，将细胞接种于96孔细胞培养板内，置于细胞培养箱内培养，细胞贴壁后，实验组加入BMP7（100 ng/mL，对照组加入等体积PBS。每过24 h每组各取6孔细胞检测增殖情况。每孔内加入20 mL MTT溶液，37 ℃孵育4 h后，吸取培养液，加入150 μL DMSO，置摇床上低速振荡10 min，使结晶物充分溶解。在酶联免疫检测仪490 nm处测量各孔的吸光值。

### 1.5 抗体阻断实验

以1×10^3^/孔的细胞密度，将细胞接种于96孔细胞培养板内，将细胞分成6组，分别为：对照组；BMP7处理组；BMP7+BMPR1A处理组；BMP7+BMPR1B处理组；BMP7+ACVR1A处理组；BMP7+BMPR1A+BMPR1B处理组。每组设6个复孔。细胞在10%胎牛血清的RPMI-1640培养液、37 ℃培养箱培养至贴壁，吸除培养液，PBS洗涤细胞2次，向BMP7+BMPR1A处理组、BMP7+BMPR1B处理组、BMP7+ACVR1A处理组、BMP7+BMPR1A+BMPR1B处理组分别加入经PBS稀释的（浓度为100 ng/mL）鼠抗人单克隆抗体BMPR1A、BMPR1B、ACVR1A、BMPR1A+BMPR1B，每孔1 mL。对照组与BMP7处理组分别每孔加入等量的PBS。抗体加入1 h后吸除，PBS洗涤2次，向各处理组中每孔加入含外源性BMP7（浓度为100 ng/mL）的上述1640培养液，对照组加入等量的上述1640培养液。每隔24 h每组各取6孔采用MTT法检测细胞的增殖情况。连续检测至第96 h。

### 1.6 统计学分析

应用SPSS 13.0统计分析软件，细胞实验结果采用*t*检验进行数据分析，*P* < 0.05为有统计学差异。

## 2 结果

### 2.1 BMPR1A、BMPR1B、ACVR1A在4种NSCLC细胞系和HBE细胞系中的表达情况

应用RT-PCR方法检测4种NSCLC细胞系和HBE细胞系中不同的Ⅰ型受体mRNA的表达情况，结果显示Ⅰ型受体的表达具有细胞系特异性。其中，在NCI-H460细胞系中三种Ⅰ型受体均有表达，在A549、LTEP-a-2细胞系中BMPR1A、BMPR1B两种Ⅰ型受体同时表达，在HBE中仅BMPR1A一种受体表达，而在SPC-A-1中则三种受体几乎均无表达（图 1）。

**1:**
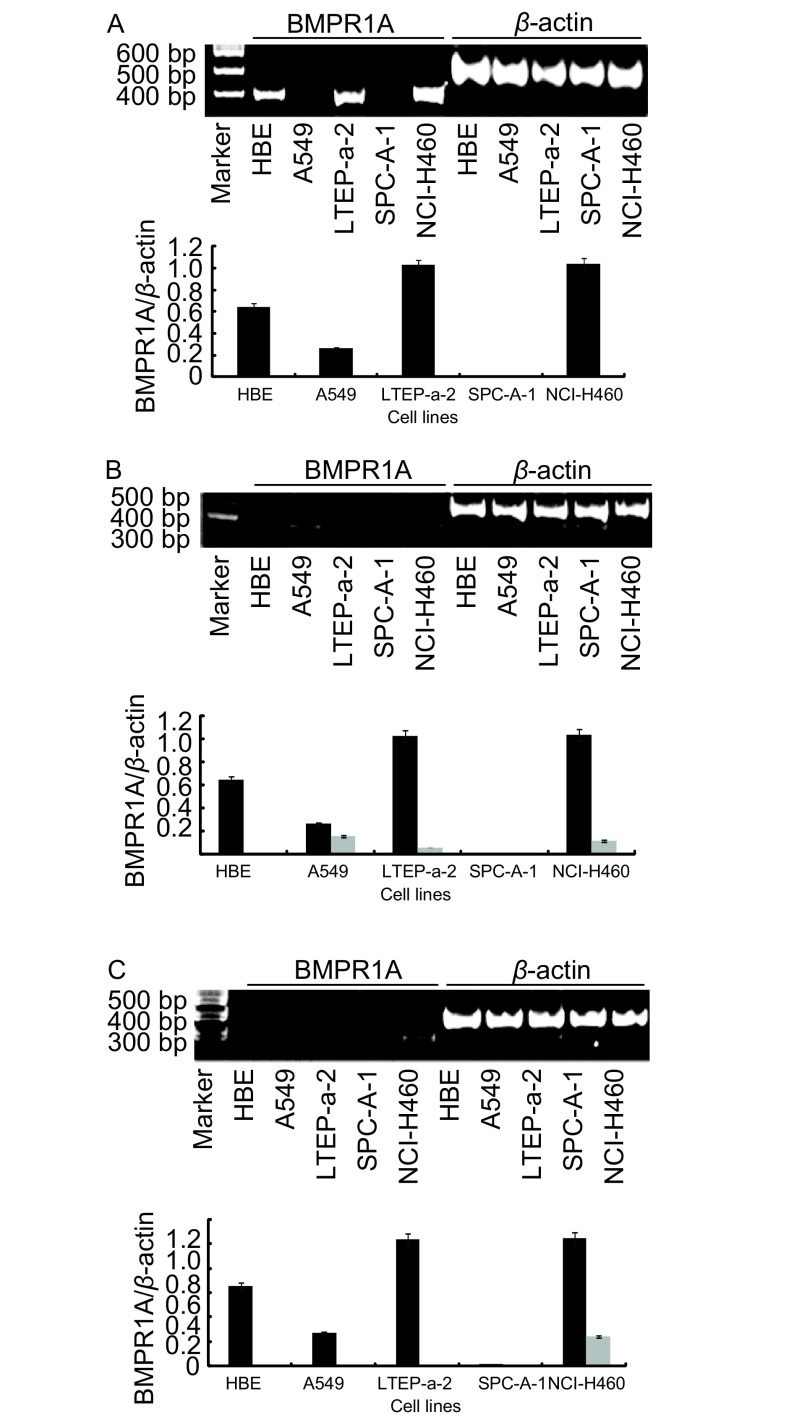
不同细胞中BMP Ⅰ型受体mRNA的表达情况。A：BMPR1A mRNA在各细胞系中的表达情况；B：BMPR1B mRNA在各细胞系中的表达情况；C：ACVR1A mRNA在各细胞系中的表达情况。 The mRNA expression levels of BMP type Ⅰ receptors. A: Expression of BMPR1A mRNA in cell lines; B: Expression of BMPR1B mRNA in cell lines; C: Expression of ACVR1A mRNA in cell lines.

### 2.2 外源性BMP7对肺大细胞癌NCI-H460细胞增殖的影响

通过上述的实验，我们筛选出同时表达三种Ⅰ型受体的NCI-H460细胞作为研究对象来研究BMP7对细胞增殖的影响。首先，我们向常规培养的NCI-H460细胞中加入外源性BMP7（20 ng/mL、50 ng/mL、100 ng/mL、150 ng/mL）并通过MTT法检测培养48 h后细胞增殖的变化情况（实验重复3次）。结果显示与对照组相比外源性BMP7具有显著抑制NCI-H460细胞增殖的能力（*P*=0.013, *P* < 0.001, *P* < 0.001, *P* < 0.001），并且其抑制细胞增殖的能力具有浓度依赖性。其中BMP7浓度 > 100 ng/mL时对细胞增殖的抑制最为显著（图 2）。随后，我们又向常规培养的NCI-H460细胞中加入外源性BMP7（100 ng/mL）并通过MTT法检测培养24 h、48 h、72 h、96 h后细胞增殖的变化情况（每组设6个重复孔，实验重复3次），结果显示，BMP7抑制细胞增殖的能力亦具有时间依赖性（*P*=0213, *P*=0.023, *P* < 0.001, *P* < 0.001），其中BMP7处理第72 h时其抑制细胞增殖的能力最为显著（图 3）。

**2:**
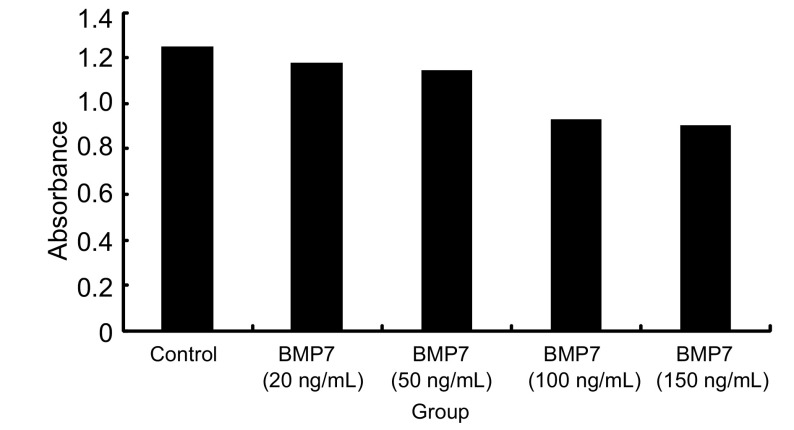
BMP7以浓度依赖方式抑制NCI-H460细胞的增殖 BMP7 inhibit proliferation of NCI-H460 cell in dosedependent manner

**3:**
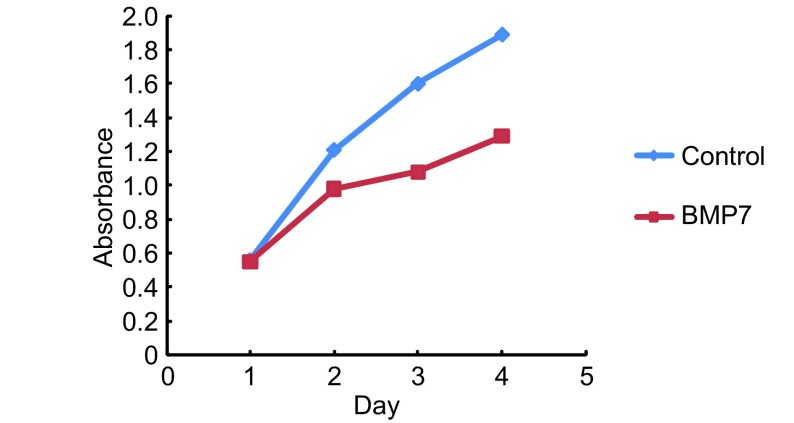
BMP7以时间依赖方式抑制NCI-H460细胞的增殖 BMP7 inhibit proliferation of NCI-H460 cell in timedependent manner

### 2.3 特异性抗体分别阻断BMPR1A、BMPR1B、ACVR1A、BMPR1A+BMPR1B后BMP7对NCI-H460细胞增殖的影响

为了进一步研究BMPR1A、BMPR1B、ACVR1A在BMP7抑制NCI-H460细胞增殖中的作用，我们向正常培养的NCI-H460细胞的培养基中分别加入浓度为100 ng/mL的特异性抗体anti-BMPR1A、anti-BMPR1B、anti-ACVR1A、anti-BMPR1A+anti-BMPR1B阻断内源性Ⅰ型受体，然后再向细胞中加入浓度为100 ng/mL的外源性BMP7（详见抗体阻断实验），通过MTT法检测96 h内细胞增殖的变化情况。实验重复3次。结果显示与阴性对照组相比anti-BMPR1A、anti-BMPR1B、anti-BMPR1A+anti-BMPR1B阻断组BMP7对细胞增殖的抑制能力明显减弱，差异有统计学意义（*P*=0.003, *P*=0.014, *P* < 0.001），其中anti-BMPR1A+anti-BMPR1B阻断组BMP7对细胞增殖的抑制能力几乎减弱至0。而anti-ACVR1A阻断组BMP7对细胞增殖的抑制能力减弱不明显，无统计学意义（*P*=0.074）。这说明BMP7主要是通过BMPR1A、BMPR1B两种Ⅰ型受体抑制NCI-H460细胞增殖（图 4，图 5）。本实验中三种抗体浓度的确定是依据系列滴度分析实验，即：将细胞接种于96孔板中并将其分成对照组、BMP7处理组、BMP7+anti-BMPR（50 ng/mL）处理组、BMP7+anti-BMPR（100 ng/mL）处理组、BMP7+anti-BMPR（150 ng/mL）处理组，每组设6个复孔。按照抗体阻断实验中的操作方法向各处理组中依次加入相应浓度的抗体及100 ng/mL的BMP7，72 h后进行MTT实验。实验重复3次，结果表明anti-BMPR1A和anti-BMPR1B对BMP7信号的阻断能力具有浓度依赖性，并且它们在浓度≥100 ng/mL时的阻断能力尤为显著。而anti-ACVR1A对BMP7信号的阻断效果不显著（图 6）。

**4:**
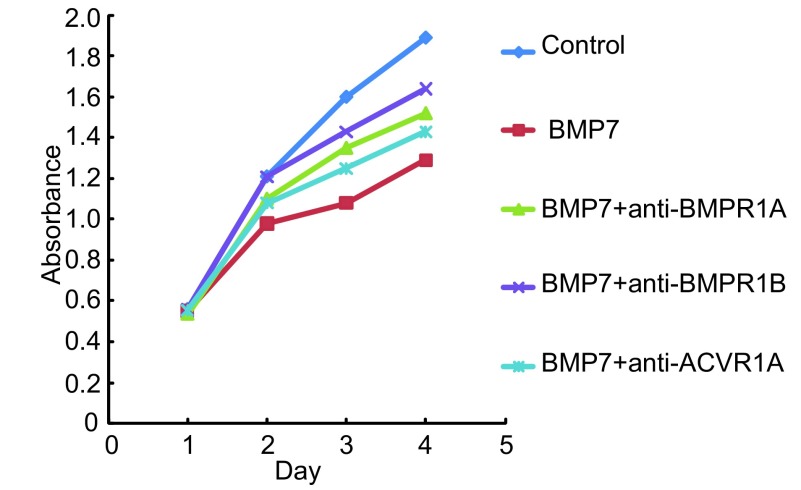
特异性抗体分别阻断BMPR1A、BMPR1B、ACVR1A后BMP7抑制NCI-H460细胞增殖能力的变化情况 The effect of BMP7 treatment on NCI-H460 cell in proliferation after bl℃king endogenous BMPR1A, BMPR1B and ACVR1A respectively

**5:**
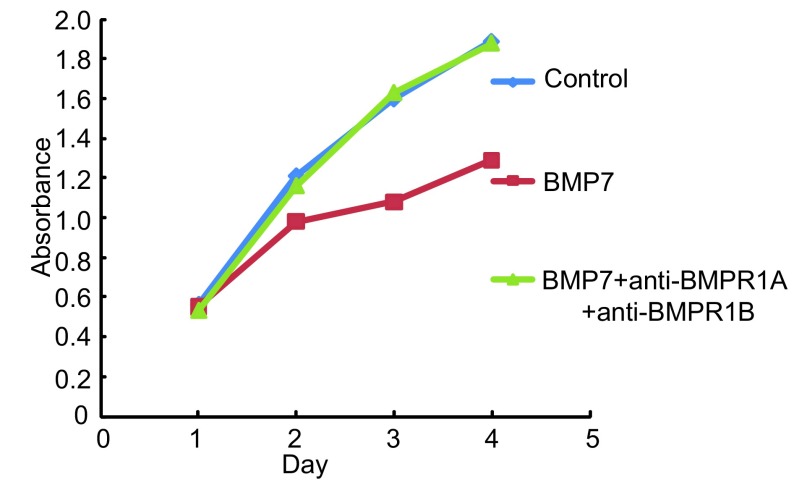
特异性抗体联合阻断BMPR1A、BMPR1B后BMP7抑制NCI-H460细胞增殖能力的变化情况 The effect of BMP7 treatment on NCI-H460 cell in proliferation after blocking endogenous BMPR1A and BMPR1B

**6:**
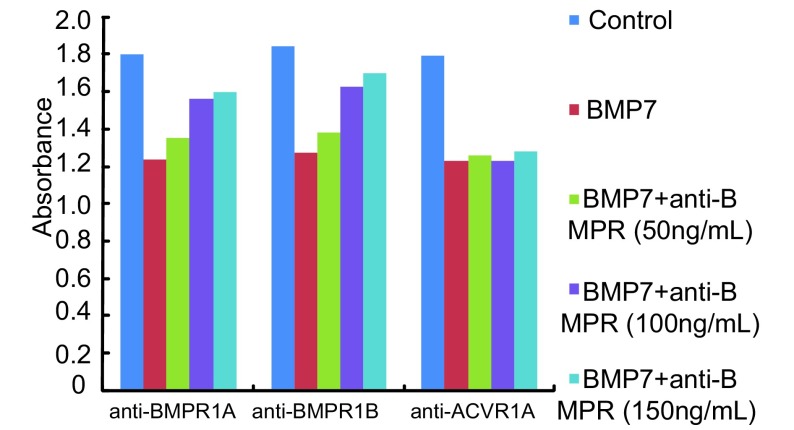
不同浓度Ⅰ型抗体阻断对BMP7抑制NCI-H460细胞增殖能力的影响情况 The effect of anti-type Ⅰ receptors at different concentrations on proliferation of NCI-H460 cell treated by BMP7

## 3 讨论

BMPs是TGF-beta超家族的成员，BMPs最初是从骨髓中被分离出并以其具有成骨作用而被人们所认识^[1]^。随着对BMPs研究的不断增多，人们发现BMPs还具有调控细胞增殖、分化、凋亡等多种生物学作用，并在胚胎发育、器官形成过程中发挥了重要的作用^[2]^。BMPs通过与靶细胞表面的受体结合而发挥作用，并且其受体与其它TGF-beta超家族成员分子的受体一样，分为Ⅰ型和Ⅱ型，属于丝-苏氨酸激酶受体家族^[3]^。其中Ⅰ型受体包括BMPR1A、BMPR1B、ACVR1A三个亚型，Ⅱ型受体包括ACVRⅡA、ACVRⅡB、BMPR2三个亚型。BMPs引起的信号传递需要与Ⅰ型、Ⅱ型受体形成复合体形式。BMPs首先与Ⅱ型受体结合随后诱导Ⅰ型受体发生磷酸化，激活的Ⅰ型受体迅速作用于细胞内相应的Smads信号传递蛋白，通过Smads蛋白启动信号转导，形成有活性的复合物，进入细胞核内，发挥相应的生物学效应^[4]^。BMPs可以结合不同的Ⅰ型受体，表现为信号传递的多样性，且Ⅰ型受体决定信号传递的特异性^[5]^。然而，另有研究证实BMPs与Ⅰ型受体的结合也具有一定的选择性，例如：BMP7倾向于与ACVR1A优先结合，同时它与BMPR1A、BMPR1B也有一定的亲和力，而BMP2、BMP4倾向于与BMPR1A结合^[6-8]^。但是，目前这三种BMP Ⅰ型受体在BMPs信号传递过程中的具体作用机制尚不明确。

目前大量的研究发现BMPs及其受体在肿瘤的发生发展过程中发挥了重要而复杂的作用。首先，BMPs及其受体的表达异常与肿瘤的发生发展密切相关。例如，Yang等^[9]^在小鼠前列腺癌模型中的研究表明：随着病情的发展，前列腺中的BMP7表达量逐渐增加。而BMPR1A、BMPR1B的表达逐渐减少甚至消失^[10]^。Rothhammer等^[11]^的研究表明BMP7在痣、恶性黑色素瘤原发灶、恶性黑色素瘤转移灶中的表达量依次增强；BMP4在痣、恶性黑色素瘤转移灶、恶性黑色素瘤原发灶中的表达量依次增强，并且内源性BMP4具有促进黑色素瘤细胞迁移和转移的作用；BMP7在恶性黑色素瘤中的表达与肿瘤的淋巴结转移和复发率呈正相关，BMP7表达可以作为恶性黑色素瘤进展的一个新的诊断指标^[12]^。Alarmo等^[13]^对大量乳腺肿瘤细胞系、乳腺癌组织、正常乳腺组织进行了RT-PCR检测，分析了BMP2-BMP8及其各种受体的表达情况，结果发现在乳腺癌细胞系中BMP4、BMP7的表达明显上调而BMPR（除外BMPR1B）的表达无显著差异。值得注意的是，BMPR1B在正常乳腺组织中无表达，而在大部分乳腺癌高表达，并且ER阳性乳腺癌中BMPR1B的表达与肿瘤的高分级、高增殖能力及不良预后呈正相关^[13, 14]^。其次，BMPs在肿瘤中发挥的生物学效应具有组织学和细胞学特异性。例如：BMP2在胰腺癌细胞中具有促进肿瘤细胞增殖的作用^[15, 16]^，而它在乳腺癌、前列腺癌LNCaP细胞、胃MKN74细胞以及正常结肠上皮细胞中却具有抑制增殖的作用^[17-19]^。另外，BMP7在前列腺BPH-1上皮细胞中具有抑制细胞增殖的作用，而在前列腺癌PC-3细胞、C4-2B细胞中却分别具有促进侵袭转移和抑制由血清饥饿诱导的细胞凋亡的作用^[19]^。综上所述，BMPs具有促进和抑制肿瘤发生发展的双重作用。然而，BMPs在肿瘤发生发展中如此复杂的生物学作用是否与BMPs分别与不同的Ⅰ型受体结合后产生不同的生物学作用有关，目前仍未见报道，BMPs对肺癌细胞的增殖有何影响目前亦未见报道。本实验基于BMP7能够与三种Ⅰ型受体结合的理论基础，筛选出了三种Ⅰ型受体均有表达的肺大细胞癌NCI-H460细胞作为研究对象，首先研究了BMP7对NCI-H460细胞增殖的作用，然后通过分别阻断三种Ⅰ型受体，研究了BMP7与不同的Ⅰ型受体结合后对NCI-H460细胞增殖的影响。结果表明BMP7通过同时激活BMPR1A、BMPR1B共同抑制NCI-H460细胞的增殖。而ACVR1A在BMP7信号传导过程中对NCI-H460细胞增殖的影响不明显。本实验结果为在前列腺癌中BMPR1A、BMPR1B表达下调提供了理论依据。值得注意的是，尽管BMPR1A、BMPR1B在BMP7信号传导过程中均具有抑制NCI-H460细胞增殖的作用，但是他们很有可能通过激活不同的信号通路共同发挥抑制细胞增殖的作用。

由于本实验涉及的细胞系非常局限，也未探讨BMP7及其Ⅰ型受体对肺癌细胞的侵袭、转移及凋亡等生物学作用的影响，亦未排除ACVR1A在蛋白水平无表达或者即使表达也无被BMP7激活的可能性，因此还不能解释ACVR1A在BMP7信号传递过程中所发挥的作用。本实验仅为今后进一步研究BMP7在肿瘤中的作用机制奠定了部分基础，也为临床治疗提供了新的可能靶点。
